# Relationship Between Visual Acuity and Cognitive Functions in Older Adults With Visual Impairment: The Mediating Role of Frailty

**DOI:** 10.1002/brb3.71113

**Published:** 2025-12-07

**Authors:** Nurcan Düzgün, Sibel Arguvanlı Çoban, Ali Ender Kulak

**Affiliations:** ^1^ Faculty of Nursing, Department of Mental Health and Disease Nursing Gazi University Ankara Türkiye; ^2^ Fethiye Faculty of Health Sciences, Department of Gerontology Muğla Sıtkı Koçman University Muğla Türkiye; ^3^ Sincan Training and Research Hospital, Department of Ophthalmology Health Sciences University Ankara Türkiye

**Keywords:** cognitive impairment, frailty, old age, visual impairment

## Abstract

**Background:**

There is a complex and interacting relationship between visual acuity, cognitive dysfunction, and frailty. It is suggested that frailty may mediate the relationship between visual acuity and cognitive impairment in older adults.

**Objectives:**

This study aimed to examine the mediating role of frailty in the relationship between visual acuity and cognitive function in older adults with visual impairment.

**Methods:**

This cross‐sectional correlational study was conducted with 116 participants with visual impairment at an ophthalmology clinic in Turkey between January and February 2025. Data were collected using an information form, the Standardized Mini‐Mental Test, and the Edmonton Frailty Scale. Visual acuity was expressed as a logMAR score, ranging from 1.00 to −0.30.

**Results:**

The mean age of the participants was 70.92 ± 5.92. The logMAR score positively and significantly associated with frailty levels (*p* < 0.001). Frailty was found to have a significant negative effect on cognitive function (*p* < 0.001). In addition, a significant negative relationship was identified between cognitive function and the logMAR score (*p* < 0.01). Both the total and direct effects of the logMAR score on cognitive function were significant (*p* < 0.001). The indirect effect, tested using the bootstrap method, was also significant (Coeff = −5.304, BootSE = 1.079, 95% CI [−7.652, −3.462]).

**Conclusions:**

These results suggest that the deterioration of visual acuity strongly impacts cognitive function and that frailty may mediate this relationship. Protecting visual health and preventing frailty in older adults may play a critical role in reducing the risk of cognitive impairment. Regular eye examinations, early intervention for vision disorders, and frailty prevention strategies can contribute to maintaining cognitive health.

## Graphical abstract

The results of this study suggest that the overall effect of visual acuity on cognitive function in older adults is strong and that the level of frailty may indirectly increase this effect.

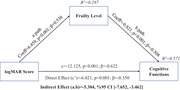

## 1 Introduction

Cognitive impairment (CI) in older adults is recognized as a significant public health problem. Studies indicate that CI is common in this population. Worldwide, the prevalence of mild CI among individuals aged 50 years and older is reported at 15.56% (Bai et al. 2022). Rates of mild CI among older adults have been reported as 62.3% in the United States (Kulshreshtha et al. 2024), 35.4% in Pakistan (Khattak et al. 2024), 19.6% in China (Wu et al. 2024), 14.7% in Nigeria (Kareem et al. 2024), and 13.3% in Europe (Rikos et al. 2024). Nationally representative data on the prevalence of CI in the elderly population in Turkey is quite limited. The overall prevalence of CI was found to be 52.1% in a study (Yavuz Veizi et al. 2024) and 18.4% in another study (Şentürk et al. 2021). However, the prevalence of visual impairment (VI) in older adults in Turkey increases significantly with age, reaching 46.5% in those over 75 years of age (Yurdakul 2023).

Growing evidence shows that VI negatively affects cognitive functioning. Previous studies have suggested older adults with VI are at greater risk for CI (Cao et al. 2023; Nagarajan et al. 2022; Varadaraj et al. 2021; Vu et al. 2021). The risk of CI is 35% higher in older adults with VI than those without VI (Shang et al. 2021). In addition, individuals with impaired visual acuity (VA), contrast sensitivity, and stereo acuity are also at increased risk for CI (Swenor et al. 2019). VI is independently associated with cognitive decline and is considered one of the modifiable risk factors for mild CI (Nagarajan et al. 2022; World Health Organization 2023).

Frailty, another modifiable risk factor, is associated with many adverse outcomes in older adults, and all older individuals are at risk for frailty (de Breij et al. 2021). Although the global prevalence of frailty remains uncertain (Hoogendijk et al. 2019), a meta‐analysis reported a prevalence of 17.4% (ranging from 3.9% to 51.4%) among individuals aged 50 years and older (Siriwardhana et al. 2018). The prevalence of frailty in older adults in Turkey ranges from 7.1% to 24.5% (Çakmur 2015; Pala and Yalçin Gürsoy 2023). Literature reviews indicate that frailty is closely related to CI (Yuan et al. 2021) and VI (Sonnenfeld et al. 2024; Kawaguchi et al. 2023). In older adults, VI is associated with the development of frailty, with those experiencing VI being more likely to develop frailty (Kawada 2021). Frailty in older adults may mediate the relationship between VI and CI, however, VI and frailty may increase the risk of subjective memory complaints (Yu and Woo 2019). Frailty in older adults may strengthen the relationship between VI and CI, thus guiding the development of strategies to prevent or delay cognitive decline (Kawada 2021; Yu and Woo 2019).

In light of this information, it can be stated that there is a strong relationship between VI, frailty, and CI. Frailty may play a mediating role in this relationship, increasing the risk of CI in older adults with VI. The relationship between visual‐ CI and frailty is intertwined, and each condition may amplify the effects of the other (Dinarvand et al. 2024). However, studies exploring this interaction are limited. No studies have been found that demonstrate this relationship. However, a study titled “Mediator Role of Frailty and Biological Deficits in Dementia Prognosis—Retrospective Cohort Study” found a significant association between frailty and the clinical course of dementia. The same study found that frailty was a complete mediator, and increasing comorbidity burden increased frailty and, consequently, CI (Işık et al. 2024). It is, therefore, essential to focus on VI and frailty as modifiable risk factors. The mechanisms explaining the relationship between VI and CI remain poorly understood. Frailty is a geriatric syndrome known to be associated with both vision loss and CI. Furthermore, frailty is a reversible and manageable, making it a critical public health issue. Examining the mediating role of frailty in this study may contribute to developing more holistic and effective intervention strategies to prevent CI in older individuals with VI.

The assessment of visual function is multidimensional, and VI covers a broad spectrum. This study focuses on the impact of VI on cognitive function in older adults in terms of visual function. Maintaining visual health and preventing frailty in older adults may be a key strategy in reducing the risk of CI. In this context, this study was conducted to determine the mediating role of frailty in the relationship between VA and cognitive function in older adults.

The aim of this study was to determine the relationship between VA and cognitive function in visually impaired older adults and to examine whether frailty mediates this relationship. The specific aims of this study were:

- To investigate the relationship between VA and cognitive function in visually impaired older adults,
- To examine the relationship between frailty and cognitive function, and
- To test whether frailty mediates the relationship between VA and cognitive function.

Based on previous evidence, the following hypotheses were formulated:

- H_1_: Lower VA will be associated with lower cognitive function in older adults.
- H_2_: Higher levels of frailty will be associated with lower cognitive function.
- H_3_: Frailty will mediate the relationship between VA and cognitive function.

## 2 Methods

### 2.1 Study Design

It is a cross‐sectional correlational study. It was conducted with individuals aged 65 and over who applied to the ophthalmology outpatient clinic of a training and research hospital in Ankara between January and February 2025.

### 2.2 Participants

The sample size was calculated with the G*Power‐3.1.9.2 program using the relevant study as a reference (De Jesus et al. 2021). For the correlation analysis, the minimum total sample size required for the study was found to be 96 with an alpha value of 0.05, an effect size of 0.58, and a theoretical power of 80%. Considering the possibility of data loss, the sample size was increased by 20% and data collection was completed when the study reached 116 participants.

The inclusion criteria were being 65 years of age or older, having one of the diseases causing VI (age‐related macular degeneration, refractive error, cataract, diabetic retinopathy, glaucoma) for at least two years, having no communication barriers, being literate, being able to speak and understand Turkish, volunteering to participate in the study, and providing written and verbal consent.

After the data collection phase was completed, a post hoc power analysis was performed using the G*Power program. In this analysis, “Visual Acuity” and “Frailty Level” were considered independent variables, and “Cognitive Function” was the dependent variable. The power analysis was based on linear multiple regression. The total sample size was 116 participants, and the model included two predictor variables. Regression analysis was performed to evaluate the effect of “Visual Acuity” and “Frailty Level” on “Cognitive Function”. The analysis yielded an *R* ^2^ value of 0.564. Based on these results, the effect size (*f* ^2^) was calculated as 1.294, with a type I error level (*α*) of 0.05. The power value of the model (1‐β) was determined to be 1.00 (100%). These findings indicate that the model had high statistical power and that the sample size was adequate for the analysis.

### 2.3 Outcome Measurements

#### 2.3.1 Descriptive Variables

Based on the literature, the researchers developed a Participant Information Form (Nagarajan et al. 2022; Varadaraj et al. 2021; Kawada 2021; Dinarvand et al. 2024), which included 18 questions evaluating the socio‐demographic characteristics of the participants.

#### 2.3.2 Cognitive Function

Cognitive function was assessed using the Standardized Mini‐Mental Test (SMMT), which was developed to evaluate CI (Güngen et al. 2002). The Turkish version of the SMMT was used in this study. This version has been tested for validity and reliability in Turkish older adult populations and has demonstrated acceptable reliability and cultural appropriateness. The SMMT provides a total score based on assessing orientation, language, attention, memory, and visual‐spatial functions. The maximum possible score is 30. A score of 24 and above indicates normal cognitive function, while a score of 23 or below indicates CI (Güngen et al. 2002).

#### 2.3.3 Frailty

Frailty was measured using the Edmonton Frailty Scale (EFS) (Aygör et al. 2018). The Turkish version of the scale was used in this study. This version has been tested for validity and reliability in Turkish older adult populations and has demonstrated acceptable reliability and cultural appropriateness. The scale consists of 11 items, with scores ranging from 0 to 17. Scores of 0–4 indicate not frailty, scores of 5–6 indicate apparently frail, scores of 7–8 indicate mild frailty, scores of 9–10 indicate moderate frailty, and scores of 11 and above indicate severe frailty. Cronbach's alpha of the EFS was reported to be 0.75 (Aygör et al. 2018). Cronbach's alpha was found to be 0.70 for this study.

#### 2.3.4 Visual Function

Data on visual function were obtained by assessing VA and refractive error. VA refers to the degree to which a pattern must differ in size to be perceived (Varadaraj et al. 2021). It was measured binocularly at a distance of 2.4 m using the Snellen threshold, with participants wearing their corrective lenses. VA was expressed as a logMAR score, ranging from 1.00 to −0.30. Lower scores indicated better VA. Refractive error was measured using an auto‐refractometer (Topcon KR‐800 Auto Keratorefractometer, Japan) operated by trained personnel. The mediation analysis only VA (logMAR) as the independent variable. Refractive error was reported to describe participant characteristics and was not included in the model. Participants were informed about the procedure and instructed to focus on the device's internal target. As stated in the user manual, when the dots and lines on the corneal display became clear, the operator pressed the button on the joystick to capture the measurement. Measurements were taken for both eyes, and the values provided by the device were recorded as the refractive error.

#### 2.3.5 Data Collection

Data were collected after obtaining ethics committee approval and institutional permission. At the beginning of the study, individuals who met the inclusion criteria were informed about the study and their informed consent was obtained. Visual function was assessed in the examination room by the ophthalmologist. A psychiatric nurse assessed frailty and cognitive function in the interview room. Since the data were collected directly by the researchers, validated instruments and standard scales were used in the measurements to minimize the risk of measurement bias. Data were collected through face‐to‐face interviews. Data collection for one participant took approximately 20–25 min.

### 2.4 Statistical Analysis

Data analysis was performed using the IBM SPSS Statistics 30 program. The Kolmogorov–Smirnov test was used to evaluate the normality of continuous dependent variables. However, since this test tends to yield significant results in large samples, visual tools such as skewness and kurtosis values, Q–Q plots, and histograms were also used to evaluate normality (Pallant 2020). Skewness and kurtosis coefficients within the range of ±2 were considered acceptable for normal distribution (George and Mallery 2024), and z‐scores within the range of ±3 were used for outlier detection (Karagöz 2023). These criteria were applied to ensure statistical objectivity regarding normality and outlier assessment. As all continuous variables met these assumptions, parametric tests were used.

Skewness and kurtosis values for the EFS, SMMT, and VA measurements were within the acceptable ±2.0 range: for EFS, skewness was 0.237 and kurtosis was −0.581; for SMMT, skewness was −0.737 and kurtosis was 0.179; for VA, skewness was 1.266 and kurtosis was 0.874. These results confirmed that the distributions of the scales were approximately normal, justifying the use of parametric tests (George and Mallery 2024).

The relationship between continuous variables was assessed using Pearson correlation analysis. The data were examined for normality, outliers, and multicollinearity to meet the assumptions for regression analysis. Normality was evaluated through skewness and kurtosis coefficients (±2.0), and outliers were identified and removed using Mahalanobis distance values. To address multicollinearity, variables with tolerance values greater than 0.2 and VIF values less than 10 were included in the analysis (Karagöz 2023). In addition, to test the mediating role of frailty in the relationship between VA and cognitive function, Model 4 proposed by (Hayes 2022) was used. This analysis was performed using the PROCESS macro v4.2 for SPSS, SAS, and R, developed by Andrew F. Hayes (2012) (Hayes 2012), available at https://www.processmacro.org/index.html. Measurement reliability for the scales was assessed using Cronbach's *α* coefficient. The level of statistical significance for all tests was set at *p* < 0.05. A regression analysis based on the bootstrap method was conducted to examine whether frailty mediated the effect of VA on participants' cognitive function.

### 2.5 Ethical Considerations

For this study, ethical approval was obtained from the Gazi University Ethics Committee (Research Approval No: 2024‐1238) and permission from the institution where the research was conducted. Participants were informed about the study, and written informed consent was obtained from those who agreed to participate.

## 3 Results

The mean age of the participants was 70.92 ± 5.92 (min = 65, max = 91). Of the participants, 62.9% were female, 73.3% were married, and 59.5% had completed primary education. Most (97.4%) were unemployed, 62.9% resided in the city center, and 72.4% lived in a nuclear family. In addition, 66.4% reported having a moderate income, and 47.4% lived only with their spouse (Table 1).

**TABLE 1:** Sociodemographic Characteristics of Participants (*n* = 116).

Quantitative characteristics		$\bar{x}$ ± SD	Min.–Max.
Age		70.92 ± 5.92	65–91
Qualitative characteristics	Subgroups	Number (*n*)	Percentage (%)
Gender	Female	73	62.9
	Male	43	37.1
Marital status	Single	31	26.7
	Married	85	73.3
Education	Literate	28	24.1
	Primary education	69	59.5
	High School∖License	19	16.4
Employment status	Unemployed	113	97.4
	Employed	3	2.6
Region of residence	City center	73	62.9
	District	30	25.9
	Town∖Village	13	11.2
Family type	Nuclear	84	72.4
	Extended	11	9.5
	Broken	21	18.1
Income status	Poor	33	28.4
	Moderate	77	66.4
	Good	6	5.2
People living together	Alone	19	16.4
	Spouse	55	47.4
	Spouse and Child	22	19.0
	Child	11	9.5
	Other relatives	9	7.8

*Note*: $\bar{x}$, mean.

Abbreviations: %, percentage; Min, minimum value; Max, maximum value; *n*, number; SD, standard deviation.

Clinical characteristics, 68.1% of the participants were independent in activities of daily living, and 89.7% had at least one chronic disease. The most common chronic condition was hypertension (68.3%). Continuous medication use was reported by 93.1% of the participants, 81.9% had undergone surgery, and 73.3% used prosthetic devices. The mean duration of glasses use was 10.64 ± 9.69 years (min = 0, max = 50), with 86.2% using glasses. The most common VI reported was cataract (53.4%) (Table 2).

**TABLE 2:** Clinical characteristics of participants (*n* = 116).

Quantitative characteristics		$\bar{x}$ ± SD	Min.–Max.
Use of glasses (years)		10.64 ± 9.69	0–50
Qualitative characteristics	Subgroups	Number (*n*)	Percentage (%)
Independence status	Semi‐dependent	37	31.9
	Independent	79	68.1
Chronic disease	No	12	10.3
	Yes	104	89.7
Types of chronic diseases^a^	Hypertension	71	68.3
	Diabetes mellitus	51	49.0
	Heart diseases	34	32.7
	Rheumatic diseases	12	11.5
	Depression	8	7.7
	COPD	7	6.7
	Osteoporosis	5	4.8
	Other	15	14.4
Continuous medication use	No	8	6.9
	Yes	108	93.1
Previous surgery	No	21	18.1
	Yes	95	81.9
Using prosthesis	No	31	26.7
	Yes	85	73.3
Types of prosthesis^a^	Dentures	78	90.7
	Walking aids	19	22.1
	Hearing aids	3	3.5
Using glasses	No	16	13.8
	Yes	100	86.2
Visual Impairment^a^	Cataract	62	53.4
	Refractive error	42	36.2
	Age‐related macular degeneration	34	29.3
	Glaucoma	15	12.9
	Diabetic retinopathy	9	7.8

*Note*: $\bar{x}$, mean; %, percentage.

Abbreviations: Max, maximum value; Min, minimum value; *n*, number, SD, standard deviation.

^a^More than one answer option (percentage values ​​are calculated based on *n* = 116).

The mean score on the EFS was 7.51 ± 3.18 (min = 0, max = 15), the score on the SMMT was 18.76 ± 5.14 (min = 4, max = 28), and the mean logMAR score for VA was 0.34 ± 0.26 (min = 0.10, max = 1.00). There was a significant negative correlation between EFS and SMMT scores (*r* = −0.696, *p* < 0.01), a significant positive correlation between EFS and logMAR scores (*r* = 0.536, *p* < 0.01), and a significant negative correlation between SMMT and logMAR scores (*r* = −0.622, *p* < 0.01). The study findings show that hypotheses H_1_ and H_2_ are supported. When the frailty levels of the participants were evaluated, it was found that 17.2% were classified as “mildly frail,” 19.0% as “moderately frail,” and 19.0% as “severely frail”. In addition, CI was found in 75.0% of the participants (Table 3).

**TABLE 3:** EFS, SMMT and LogMAR score and correlation analysis.

Measurement tools	$\bar{x}\;$± SD	Min.‐Max.	1	2	3
1‐ EFS	7.51 ± 3.18	0–15	—		
2‐ SMMT	18.76 ± 5.14	4–28	−0.696**	—	
3‐ LogMAR	0.34 ± 0.26	0.10–1.00	0.536**	−0.622**	—

**Frailty score**	**Number (n)**	**Percentage (%)**	**Description**
0–4	21	18.1	Not frail
5–6	31	26.7	Apparently frail
7–8	20	17.2	Mildly frail
9–10	22	19.0	Moderately frail
11 and above	22	19.0	Severely frail
**Cognitive Function Score**			
24 and above	29	25	Normal cognitive function
23 and below	87	75	Cognitive impairment

*Note*: $\bar{x}$, mean, %, percentage,

Abbreviations: Max, maximum value; Min, minimum value; *n*: Number; SD, standard deviation.

**p < 0.01.

**FIGURE 1:**
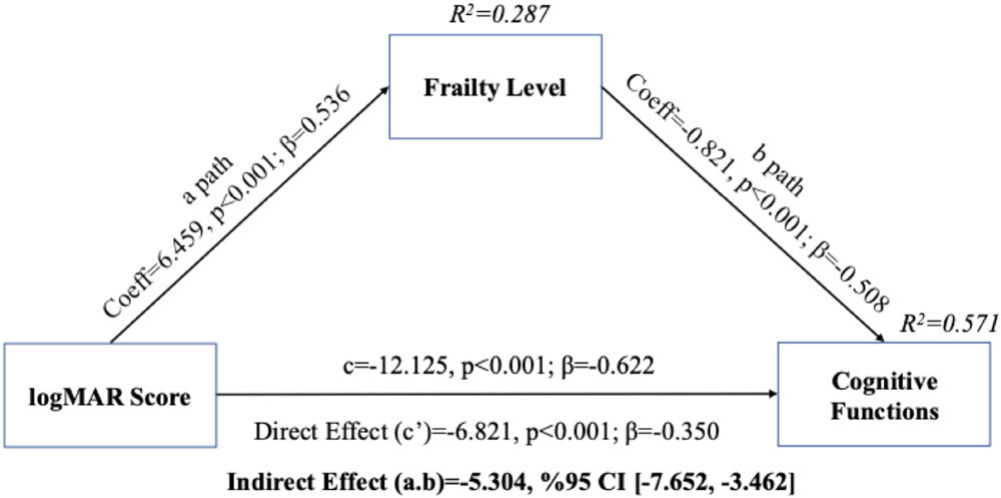
Mediating effects of frailty on the relationship between logMAR score and cognitive functions.

In this study, the logMAR score was found to have a significant positive effect on frailty levels (Coeff = 6.459, SE = 0.953, *β* = 0.536, *p* < 0.001). The analysis also revealed that the level of frailty had a significant negative effect on cognitive function (Coeff = −0.821, SE = 0.118, *β* = −0.508, *p* < 0.001). Furthermore, the direct impact of the logMAR score on cognitive function was significant (Coeff = −6.821, SE = 1.422, *β* = −0.350, *p* < 0.001) (Figure 1; Table 4). H_3_ hypothesis was supported according to the study findings. Moreover, the total effect of the logMAR score on cognitive function (Coeff = −12.125, SE = 1.428, *β* = −0.622, *p* < 0.001) and its direct effect were significant (Coeff = −6.821, SE = 1.422, *β* = −0.350, *p* < 0.001). The indirect impact, tested using the bootstrap method, was also significant (Coeff = −5.304, BootSE = 1.079, 95% CI [−7.652, −3.462]) (Figure 1; Table 4).

**TABLE 4:** The Mediating Role of Frailty Level in the Relationship Between LogMAR Score and Cognitive Function in Older Adults.

	Outcome variables							
	*M* (Mediator variable)				*Y* (Dependent variable)			
	(EFS; Frailty level)				(SMMT; Cognitive function)			
**Prediction variables**		**Coeff**.	**SE**	***β***		**Coeff**.	**SE**	***β***
***X* (LogMAR)**	*A*	6.459*	0.953	0.536	*c’*	−6.821*	1.422	−0.350
***M* (EFS)**	—				*b*	−0.821*	0.118	−0.508
**Stable**	*i_M_*	5.287*	0.413		*i_Y_*	27.271*	0.812	
	*R* ^2^ = 0.287				*R* ^2^ = 0.571			
	*F* (1, 114) = 45.921, *p *< 0.001				*F* (2, 113) = 75.268, *p *< 0.001			
	**Coeff**.	**SE**	***β***	***p***	**LLCI**		**ULCI**	
Total effect (path c)	−12.125*	1.428	−0.622	*p* < 0.001	−14.955		−9.295	
Direct effect (c’ path)	−6.821*	1.422	−0.350	*p* < 0.001	−9.637		−4.005	
	**Coeff**.		**BootSE**.		**BootLLCI**		**BootULCI**	
**Indirect effect (a.b path)**	−5.304		1.079		**−7.652**		**−3.462**	

*Statistically significant result at *p* < 0.001 level.

SE: Standard Error, Standardized (β) and unstandardized (Coeff) beta coefficients are reported together.

LLCI (Lower Level Confidence Interval): Lower confidence interval limit, ULCI (Upper Level Confidence Interval): Upper confidence interval limit. Boot: Values ​​calculated using the bootstrap method.

## 4 Discussion

In this study, which aimed to determine the mediating role of frailty in the relationship between VA and cognitive function in older adults, it was found that the total effect of VA on cognitive function was strong, and the level of frailty may indirectly enhance this effect (Figure 1; Table 4). In the 2024 report by the Lancet Dementia Commission, which evaluated systematic reviews and meta‐analyses conducted since 2020, untreated visual loss was identified as one of the 14 major modifiable risk factors for dementia (Livingston et al. 2024). However, the underlying mechanisms linking VI and CI remain unclear (Zheng et al. 2024), highlighting the need to explore the pathways that explain this association. Previous research has investigated the relationship between VI and cognitive function and various mediating factors (Moon et al. 2024; Khalaila et al. 2024; Matthews et al. 2024; Wilson et al. 2024; Ge et al. 2019). Findings indicate that allostatic load, social interaction, depression and physical activity (Matthews et al. 2024), loneliness and low social activity (Moon et al. 2024), social support (Ge et al. 2019), and social engagement may mediate this relationship (Wilson et al. 2024). In addition, Kawada (2021) suggested that frailty may mediate the relationship between VI and CI, proposing that VI could influence CI through frailty (Kawada 2021). We anticipate that this study is the first to examine the mediating effect of frailty on the relationship between VI and cognitive function. Future studies investigating mediators of the relationship between vision and cognition may contribute to a better understanding of the underlying mechanisms. Frailty may mediate the relationship between VI and cognitive decline through multiple pathways. Factors contributing to frailty in older adults with VI may increase susceptibility to cognitive decline. This complex mechanism suggests a biopsychosocial link between sensory deficits and cognitive outcomes. We believe that conducting similar studies in diverse populations is essential to strengthen the evidence base. Given the intertwined relationship between VI, CI, and frailty, and the potential for each condition to influence the others (Kawada 2021; Dinarvand et al. 2024). It is crucial to further investigate this relationship in the future. In this study, a significant negative correlation was found between logMAR score and cognitive function (*r* = −0.622, *p* < 0.01) (Table 3). As stated in the methodology, higher scores on the SMMT indicate better cognitive function, while lower LogMAR scores reflect better VA. Accordingly, better VA was significantly associated with higher cognitive function. Moreover, both the total and direct effects of the logMAR score on cognitive function were significant (*p* < 0.001) (Table 4; Figure 1). Several studies have reported an association between VI and CI, including an increased risk of mild CI or dementia (Swenor et al. 2019; Ward et al. 2018; Loughrey et al. 2018; Chen et al. 2017). Parada et al. (2021) found that low VA was linked to decreased performance on the Mini‐Mental State Examination and a more rapid decline in cognitive function with aging (Parada et al. 2021). Another study reported that impairment in VA significantly predicted multidomain cognitive decline (Wilson et al. 2024). It has also been noted that VI may reduce cognitive function (Kawada 2021). However, it has been suggested that cognitive decline may be mitigated by addressing treatable causes such as refractive errors and cataracts (Kawada 2021). Older adults included in this current study had been under ophthalmologic follow‐up for VI for at least two years. Most participants wore spectacles, with a mean duration of spectacle use of 10.64 ± 9.69 years, and about half had cataracts (53.4%) (Table 2). The high rate of CI and frailty found in the participants in our study may be a finding indicating that this relationship is strong (Table 3). In addition, the mean logMAR score for the participants in this study was 0.34 ± 0.26. This value indicates that the overall decrease in VA was low.

These findings suggest that early intervention and prevention efforts are valuable. This is particularly relevant given that most participants in this study exhibited negative outcomes in terms of frailty and cognitive function. These results may be attributed to low educational levels and the presence of at least one VI (Tables 1, 2, 3). Future research is recommended to explore the role of early diagnosis of VI and the use of spectacles in preventing visual and cognitive decline in older adults. VI is associated with CI, which increases the risk of frailty (Ho et al. 2020; Nagarajan et al. 2020). In this study, a significant positive correlation was found between the logMAR score and frailty (*r* = 0.536, *p* < 0.01) (Table 3), indicating that frailty levels increased as VA decreased among older adults. The direct effect of the logMAR score on frailty was also significant (*p* < 0.001) (Figure 1; Table 4). In line with our findings, Swenor et al. (Varadaraj et al. 2021) reported that a one‐line worsening in VA was associated with increased odds of frailty. Several studies have identified VI as an important risk factor for the development of frailty (Kawada 2021; Swenor et al. 2019; Rodriguez‐Laso et al. 2021; Liljas et al. 2017). Individuals with VI are more likely to experience frailty than those without VI (Sonnenfeld et al. 2024; Swenor et al. 2019; Liljas et al. 2017). VI has been reported to impact several components of frailty, including reduced physical activity, slowness, fatigue, and weakness (Kawada 2021; Gonzales‐Turín et al. 2021). Moreover, beyond general VI, near vision impairment has also been associated with the severity of frailty (Kawada 2021). However, it is important to note that studies examining the relationship between vision and frailty using objective vision measurements remain limited. More research is needed to clarify this relationship's mechanisms (Swenor et al. 2019). In this study, visual function was assessed objectively by an ophthalmologist in the ophthalmology outpatient clinic using standardized diagnostic equipment. The findings of our study demonstrate that frailty plays a significant mediating role in the relationship between VA and CI. VA, cognitive function, and frailty are mutually influencing components in older adults. In geriatric care, maintaining the function of these three components, monitoring them through regular screening, and early diagnosis and management are crucial for the health of older adults. Given the mediating role of frailty in this study, multicomponent interventions designed for this purpose (such as physical exercise, nutritional support, and social participation) may provide multidimensional benefits by reducing frailty in older adults. These findings highlight the importance of multidisciplinary and preventative approaches in healthcare for older adults.

## 5 Conclusion

In conclusion, the results of this study supported all three hypotheses. Specifically, lower VA was associated with poorer cognitive function (H_1_), higher levels of frailty were linked to reduced cognitive performance (H_2_), and frailty mediated the relationship between VA and cognition (H_3_). These findings suggest that the overall effect of VA on cognitive function in older adults is strong and that the level of frailty may indirectly increase this effect. Maintaining visual health in older adults may play a critical role in reducing both frailty and the risk of CI. The Lancet Dementia Commission emphasizes the importance of addressing modifiable risk factors and recommends explicitly making vision screening and treatment accessible to all as one of the key actions across 14 identified risk factors (Aygör et al. 2018). Based on the results of this study, community‐based screening and follow‐up programs to assess visual function and frailty may be recommended. Collaborative approaches involving primary care providers are essential for this goal. In addition, community‐based screening programs that assess vision, frailty, and cognition can serve as a proactive strategy to identify at‐risk individuals early. Follow‐up services, rehabilitation support, and health education for older adults with visual impairments can also help maintain independence and improve quality of life.

### 5.1 Limitations

One limitation of the present study is that covariates such as age, gender, and education levels were not included in the mediation model. While these factors may influence both frailty and cognitive functioning, our primary aim was to explore the mediating role of frailty in the association between VA and cognition. Future research with larger samples should investigate whether the observed relationships remain significant after adjusting for potential confounders. This study was limited to Turkish older adults who presented to ophthalmology outpatient clinics with at least one form of visual impairment.

## Author Contributions

**Nurcan Düzgün**: conceptualization, methodology, writing–original draft, data curation, writing – review and editing. **Sibel Arguvanlı Çoban**: conceptualization, writing–original draft, writing–review and editing, statistical analysis. **Ali Ender Kulak**: methodology, data curation.

## Funding

The authors have nothing to report.

## Conflicts of Interest

The authors declare no conflicts of interest.

## Data Availability

The data supporting this study's findings are available from the corresponding author upon reasonable request.

## Participant Information Form

1. Age: ……………….
2. Gender:    (   ) Female     (   ) Male
3. Marital status:  (   ) Single     (   ) Married     (   ) Other (please specify)…………
4. Education:

(   ) Literate
(   ) Primary education
(   ) High School
(   ) Bachelor degree
(   ) Postgraduate

1. Employment status:   (   ) Not working   (   ) Working
2. Region of residence:   (   ) City  (   ) District    (   ) Town∖Village
3. Family type:    (   ) Nuclear family   (   ) Extended family   (   ) Broken family
4. Income status :   (   ) Poor    (   ) Middle   (   ) Good
5. People living together:

(   ) Alone
(   ) Wife
(   ) Child
(   ) Spouse and Child
(   ) Other relatives
(   ) Institution

1. Independence status:  (   ) Dependent    (   ) Semi‐dependent   (   ) Independent
2. Visual Impairment:

(   ) Age‐related macular degeneration
(   ) Refractive error
(   ) Cataract
(   ) Diabetic retinopathy
(   ) Glaucoma

1. Chronic disease: (   ) No     (   ) Yes
2. Types of chronic diseases:

(   ) Hypertension
(   ) Diabetes mellitus
(   ) Heart diseases
(   ) COPD
(   ) Rheumatic diseases
(   ) Osteoporosis
(   ) Depression
(   ) Other

1. Continuous use of medication:    (   ) No    (   ) Yes
2. Be operated:    (   ) No    (   ) Yes
3. Using prosthesis:   (   ) No   (   ) Yes
4. Types of prosthesis:

(   ) Walking aids
(   ) Dentures
(   ) Hearing aids
(   ) Other

1. Glasses usage status:   (   ) No   (   ) Yes
2. Snellen threshold measurement result:
3. Autorefractometer measurement result:

## Edmonton Frail Scale

**Table:**

Frailty domain	Item	0 point	1 point	2 point
Cognition	1. Please imagine that this pre‐drawn circle is a clock. I would like you to place the numbers in the correct positions then place the hands to indicate a time of ‘ten after eleven’.	No errors	Minor spacing errors	Other errors
General health status	2. In the past year, how many times have you been admitted to a hospital?	0	1‐2	>2
	3. In general, how would you describe your health?	Excellent Very good Good	Fair	Poor
**Functional independence**	4. With how many of the following activities do you require help? (meal preparation, shopping, transportation, telephone, housekeeping, laundry, managing money, taking medications)	0‐1	2‐4	5‐8
**Social support**	5. When you need help, can you count on someone who is willing and able to meet your needs?	Always	Sometimes	Never
**Medication use**	6. Do you use five or more different prescription medications on a regular basis?	No	Yes	
	7. At times, do you forget to take your prescription medications?	No	Yes	
**Nutrition**	8. Have you recently lost weight such that your clothing has become looser?	No	Yes	
**Mood**	9. Do you often feel sad or depressed?	No	Yes	
**Kontinans**	10. Do you have a problem with losing control of urine when you don't want to?	No	Yes	
**Functional performance**	11. I would like you to sit in this chair with your back and arms resting. Then, when I say ‘GO’, please stand up and walk at a safe and comfortable pace to the mark on the floor (approximately 3 m away), return to the chair and sit down’.	0‐10 s	11‐20 s	One of >20 s patient unwilling, or requires assistance
**Totals**	Final score is the sum of column totals			

## Standardized Mini‐Mental State Examination (SMMSE)

### Orientation (Total points: 10) (1 point for each correct answer)

What year is this?

What season is this?

What month is this?

What is today's date?

What day of the week is this?

What country are we in?

What province are we in?

What city/town are we in?

What is the name of this building?

What floor of the building are we on?

### Registration

I am going to name three objects. When I am finished, I want you to repeat them. Remember what they are because I am going to ask you to name them again in a few minutes. (Say the following words slowly at approximately one‐second intervals): Table / Flag / Dress

### Attention and Calculation (Total points: 5)

Subtract 7 from 100 and continue until I say stop.

“100, 93, 86, 79, 72, 65” (1 point for each correct number).

### Language (Total points: 9)

What are the names of these objects you see?

- “Clock, pen” (20 s, 1 point for each correct object, Total points: 2)
- Listen carefully to the sentence I'm going to tell you now and repeat it after I finish. “If and but I don't want to” (10 s, 1 point)
- Now I'm going to ask you to do something. Listen carefully and do as I say. “Please take the paper on the table with your right/left hand, fold it in half with both hands, and place it on the floor.” (30 s are allotted, (1 point for each correct answer, Total points: 3)
- Now I'll give you a sentence. Read it and do what the text says. The text “CLOSE YOUR EYES” will be shown. (1 point for correct answer)
- Now write a meaningful sentence that comes to your mind on the paper I will give you. (1 point for correct answer)
- Draw the same shape as I show you. (1 point for correct answer)

**“Close your eyes”**
